# Enrichment of intestinal *Lactobacillus* by enhanced secretory IgA coating alters glucose homeostasis in *P2rx7*^−/−^ mice

**DOI:** 10.1038/s41598-019-45724-9

**Published:** 2019-06-27

**Authors:** Lisa Perruzza, Francesco Strati, Giorgio Gargari, Anna Maria D’Erchia, Bruno Fosso, Graziano Pesole, Simone Guglielmetti, Fabio Grassi

**Affiliations:** 1 Institute for Research in Biomedicine, Faculty of Biomedical Sciences, Università della Svizzera Italiana (USI), 6500 Bellinzona, Switzerland; 2 Department of Food, Environmental, and Nutritional Sciences (DeFENS), Università degli Studi di Milano, 20133 Milan, Italy; 3 Institute of Biomembranes and Bioenergetics, National Research Council, 70126 Bari, Italy; 4 Department of Biosciences, Biotechnologies and Biopharmaceutics, University of Bari, 70126 Bari, Italy; 5 Department of Medical Biotechnology and Translational Medicine, Università degli Studi di Milano, 20129 Milan, Italy; 6 Istituto Nazionale Genetica Molecolare “Romeo ed Enrica Invernizzi”, 20122 Milan, Italy

**Keywords:** Microbiology, Immunology, Mucosal immunology, Microbiome

## Abstract

The secretory immunoglobulin A (SIgA) in mammalian gut protects the organism from infections and contributes to host physiology by shaping microbiota composition. The mechanisms regulating the adaptive SIgA response towards gut microbes are poorly defined. Deletion of *P2rx7*, encoding for the ATP-gated ionotropic P2X7 receptor, leads to T follicular helper (Tfh) cells expansion in the Peyer’s patches (PPs) of the small intestine, enhanced germinal centre (GC) reaction and IgA secretion; the resulting alterations of the gut microbiota in turn affects host metabolism. Here, we define gut microbiota modifications that correlate with deregulated SIgA secretion and metabolic alterations in *P2rx7*^−/−^ mice. In particular, *Lactobacillus* shows enhanced SIgA coating in *P2rx7*^−/−^ with respect to wild-type (WT) mice. The abundance of SIgA-coated lactobacilli positively correlates with Tfh cells number and body weight, suggesting *Lactobacillus*-specific SIgA response conditions host metabolism. Accordingly, oral administration of intestinal *Lactobacillus* isolates from *P2rx7*^−/−^ mice to WT animals results in altered glucose homeostasis and fat deposition. Thus, enhanced SIgA production by P2X7 insufficiency promotes *Lactobacillus* colonization that interferes with systemic metabolic homeostasis. These data indicate that P2X7 receptor-mediated regulation of commensals coating by SIgA is important in tuning the selection of bacterial taxa, which condition host metabolism.

## Introduction

The intestinal microbiota influences host physiology, metabolism and immune system homeostasis^1^. After birth, microbial colonization stimulates the development of host’s gut-associated lymphoid tissues (GALT), including cryptopatches, Peyer patches (PPs), and isolated lymphoid follicles^2^. The ensuing interaction between microbes and immune system results in the relative immune tolerance of the commensal microbial community and selection of beneficial taxa^3^. The secretory immunoglobulin A (SIgA) contributes to the establishment of the intestinal barrier by controlling binding of bacteria to the epithelium and the possible translocation of pathobionts into the lamina propria^4^. However, it is not yet completely understood which members of the gut microbiota are actually targeted by SIgA and which contribution IgA-coated bacteria might provide to host physiology. Although SIgA-coated bacteria have been described to be enriched in taxa with potential pro-inflammatory properties conferring susceptibility to colitis^5^ and weight loss^6^, IgA-coated bacteria from healthy humans protect mice from disease^6^ and are important for the preservation of commensal diversity and community networks in the human gut^7^. Recently, a regulatory mechanism whereby SIgA would foster mucosal colonization of the human gut commensal *Bacteroides fragilis* has been described^8^. Furthermore, SIgA-coated *Bacteroides thetaiotaomicron* induced the expression of Mucus-Associated Functional Factors (MAFFs) that regulated the composition and metabolic function of the whole gut microbiota by promoting symbiosis with members of the phylum *Firmicutes* and colonic homeostasis^9^. The vast majority of SIgA-coated bacteria resides in the small intestine and is targeted by T cell-independent antibodies; only a minority of commensals would be responsible for eliciting T cell-dependent SIgA responses^10^. T follicular helper (Tfh) cells in PPs are essential for SIgA affinity maturation that in turn modulates the structure and function of the intestinal microbiota^11^. Adenosine triphosphate (ATP) is an ubiquitous extracellular messenger, which activates purinergic receptors in the plasma membrane of eukaryotic cells termed P2X and P2Y receptors^12^. The ATP-gated ionotropic receptor P2X7 is a signature gene of effector T cell subsets^13,14^ and is selectively upregulated in Tfh cells of PPs. In mice with deletion of the *P2rx7* gene, Tfh cells are expanded in PPs because of resistance to cell death induced by extracellular ATP (eATP). The altered control of Tfh cells by defective sensing of microbiota derived ATP leads to enhanced secretion of T cell dependent IgA and increased frequency of replacement mutations in the IgV_H_1 family’s complementarity determining region (CDR) 2 suggesting enhanced affinity maturation of IgA responses^15^. Therefore, eATP modulates adaptive IgA responses to ensure physiological mucosal colonization. Furthermore, the alteration of the gut microbiota due to the lack of P2X7 mediated control of Tfh cells results in dysregulated metabolic homeostasis, consistent with the central role of SIgA in regulating host-microbiota interactions and host physiology^16^. A number of studies in mice and humans have demonstrated that obesity is associated with alterations of the gut microbiota. Intestinal dysbiosis has been suggested to play a causal role in the development of insulin resistance^17^ as well as inflammation and macrophage accumulation in adipose tissue^18^. Recently, a genome-wide association study has shown the association of hypo-functioning P2X7 variants with impaired glucose homeostasis and obesity in humans^19^. Here, the characterization of the faecal microbiota targeted by SIgA in *P2rx7*^−/−^ mice allowed us to identify the enhanced SIgA coating of *Lactobacillus* as a possible mechanism contributing to the observed metabolic disturbance.

## Results

### *P2rx7*^−/−^ mice show altered metabolic parameters and enhanced Tfh cells activity

**Figure 1:**
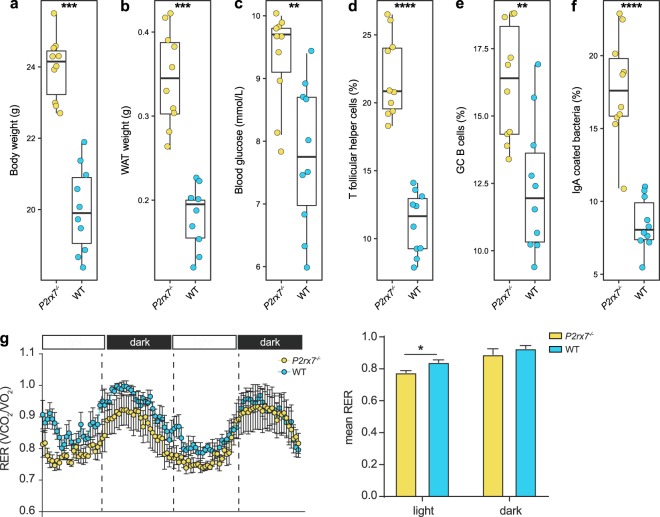
Alteration of metabolic and immunological parameters in *P2rx7*^−/−^ mice. (**a**) Body weight, (**b**) WAT weight, (**c**) blood glucose, (**d**) % of Tfh cells in PPs, (**e**) % of GC B cells in PPs, (**f**) % of faecal IgA-coated bacteria in *P2rx7*^−/−^ (yellow) and WT (blue) mice. Box plots are defined by the 25th and 75th percentiles. Centre line represents the median (50th percentile). Whiskers are defined as 1.5 times the interquartile range from the 25th or 75th percentiles. (**g**) Dynamic pattern of respiratory exchange ratio (RER) and relative mean RER values during the light and dark phase in WT and *P2rx7*^−/−^ mice (the displayed experiment is representative of three). *p < 0.05, **p < 0.01, ***p < 0.001, **** *p* < 0.0001, Wilcoxon rank-sum test; n = 10 per group.

*P2rx7*^−/−^ mice are characterized by altered fat distribution^20^. In these mice, dysregulated Tfh cells activity with consequent enhanced GC reactions and secretion of high affinity IgA affects microbiota composition resulting in altered glucose homeostasis and fat deposition^15,16^. White adipose tissue (WAT), body weight and blood glucose were increased in *P2rx7*^−/−^ mice with respect to WT littermates (*p* < 0.01, Wilcoxon rank-sum test; Fig. 1a–c). Food consumption and energy harvesting were not different between the two strains of mice. However, the daily profile of fuel metabolism, as measured by the respiratory exchange ratio (RER), the ratio of consumed oxygen to produced carbon dioxide, showed differences between the two groups of mice. *P2rx7*^−/−^ mice showed lower RER values during the inactive (light) phase with respect to WT littermates, suggesting lower energy expenditure might at least partially contribute to the body weight increase in these mice (Fig. 1g). As expected, the proportions of Tfh and GC B cells were significantly higher in *P2rx7*^−/−^ mice than in WT littermates (*p* < 0.01, Wilcoxon rank-sum test; Fig. 1d,e) as well as the percentage of IgA-coated bacteria (*p* < 0.0001, Wilcoxon rank-sum test; Fig. 1f).

### *P2rx7*^−/−^ mice harbour an altered gut microbiota and enhanced IgA response towards bacteria residing in the small intestine

**Figure 2:**
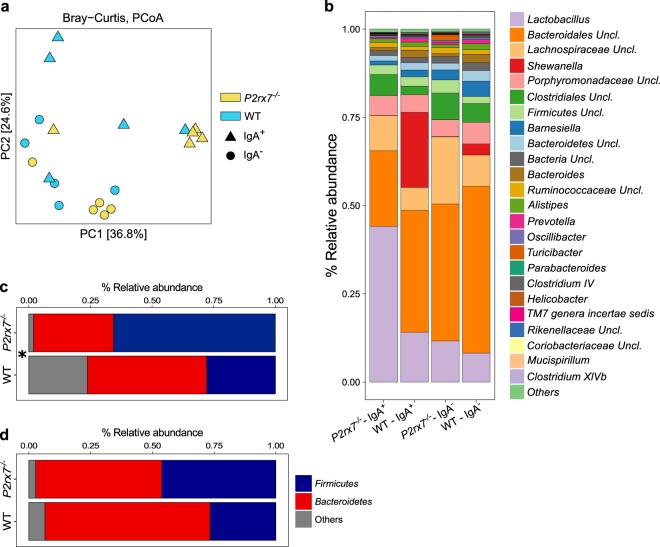
Microbial community structure of the IgA positive (IgA^+^) and IgA negative (IgA^−^) fractions of the faecal microbiota in *P2rx7*^−/−^ and WT mice. (**a**) PCoA of bacterial *beta*-diversity based on the Bray-Curtis dissimilarity. *P2rx7*^−/−^ and WT mice are coloured in yellow and blue, respectively. IgA^+^ and IgA^−^ samples are indicated as triangles and circles, respectively. (**b**) Mean relative abundances (%), at genus level, of the IgA^+^ and IgA^−^ fractions of faecal microbiota from *P2rx7*^−/−^ and WT mice. All bacterial genera with relative abundance <0.1% are reported together and labelled as “others”. (**c**,**d**) Mean relative abundances (%) of *Firmicutes* and *Bacteroidetes* in the IgA^+^ (**c**) and IgA^−^ (**d**) fractions of faecal microbiota from *P2rx7*^−/−^ and WT mice. The total abundance of all other phyla is reported as “others”. *p < 0.05, Wilcoxon rank-sum test calculated on the *Firmicutes*/*Bacteroidetes* ratio.

**Figure 3:**
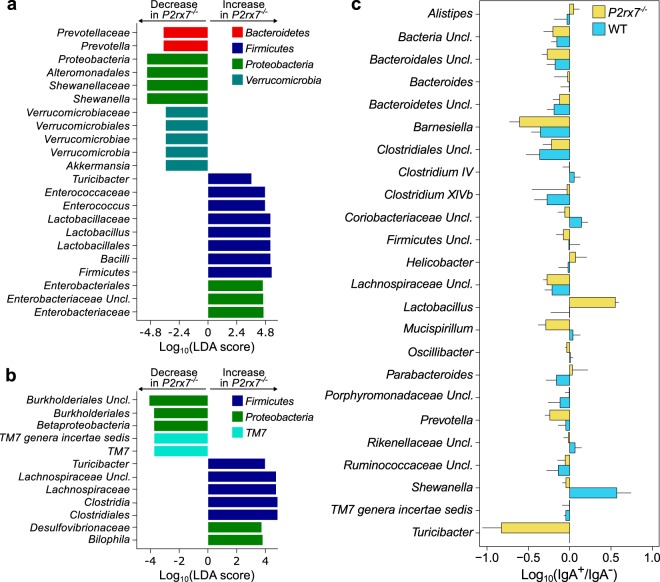
The lack of P2X7 receptor alters the gut microbiota at different taxonomic levels. (**a**,**b**) Log_10_ of LDA scores for the most discriminant bacterial taxa identified by LEfSe in the IgA^+^ (**a**) and IgA^−^ (**b**) fractions of faecal microbiota from *P2rx7*^−/−^ and WT mice. Positive and negative LDA scores indicate the taxa enriched or depleted in *P2rx7*^−/−^ mice. Only taxa having a *p* < 0.05 (Wilcoxon rank-sum test) and LDA > |2.0| are shown. (**c**) Enrichment of indicated taxa (with relative abundance >0.1%) in the IgA^+^ and IgA^−^ fractions of *P2rx7*^−/−^ mice and WT littermate controls. Error bars indicate the standard error.

In order to define possible differences in bacterial IgA coating between *P2rx7*^−/−^ mice and WT littermates, we characterized the IgA^+^ and IgA^−^ fractions of the faecal microbiota through high-throughput sequencing of the V5–V6 region of the 16S rRNA gene (IgA-SEQ) (Fig. S1). The analysis of *alpha*-diversity (*i*.*e*. the within samples diversity) revealed no significant differences between the IgA^+^ or IgA^−^ fractions of *P2rx7*^−/−^ and WT mice. Nevertheless, we observed a reduction close to statistical significance, of *alpha*-diversity in the presorted faecal samples from *P2rx7*^−/−^ mice compared to WT controls (*p* = 0.055 on the Inverse Simpson index; Wilcoxon rank-sum test), in agreement with previous observations on the gut microbiota of obese mice and humans^21,22^. We then characterized the microbial community structure of the IgA^+^, IgA^−^ and presorted faecal microbiota through *beta*-diversity analysis on the unweighted UniFrac distance and Bray-Curtis dissimilarity. The faecal microbiota of *P2rx7*^−/−^ mice clustered apart from that of WT littermates as well as the IgA^+^ fraction (*p* < 0.05, PERMANOVA on the Bray-Curtis dissimilarity, Table 1 and Fig. 2a), suggesting that enhanced IgA secretion due to lack of Tfh cells control via P2X7 has a significant effect on the composition of the gut microbiota (Figs 2b and S2a). Phylum level analysis showed a significant increase in the *Firmicutes*/*Bacteroidetes* ratio in the *P2rx7*^−/−^ IgA^+^ microbiota (*p* = 0.03, Wilcoxon rank-sum test, Fig. 2c) due to the significantly higher relative abundance of *Firmicutes* (mean relative abundance, 65.6% in *P2rx7*^−/−^, 27.8% in WT), as confirmed by LEfSe analysis (Fig. 3a). On the contrary, we did not detect significant differences in the *Firmicutes*/*Bacteroidetes* ratio of the IgA^−^ microbiota (Fig. 2d) and presorted faecal microbiota of *P2rx7*^−/−^ and WT mice (Fig. S2b). The *Firmicutes*/*Bacteroidetes* ratio is a rough estimator of intestinal dysbiosis and its increase has been associated to obesity and metabolic abnormalities in humans and mice^21,22^.

**Table 1:** Permutational multivariate analysis of variance (PERMANOVA) of the IgA^+^, IgA^−^ and pre-sorted faecal microbiota in WT *vs P2rx7*^−/−^ mice according to the unweighted UniFrac distance and Bray-Curtis dissimilarity.

	Metric	F	R^2^	*p-value*
IgA^+^	Unweighted Unifrac	1.517	0.159	0.175
WT *vs P2rx7*^−/−^	Bray-Curtis	2.611	0.246	0.046
IgA^−^	Unweighted Unifrac	1.259	0.135	0.197
WT *vs P2rx7*^−/−^	Bray-Curtis	2.220	0.217	0.054
Pre-sorted	Unweighted Unifrac	1.285	0.138	0.005
WT *vs P2rx7*^−/−^	Bray-Curtis	1.765	0.180	0.043

The enhanced SIgA response due to lack of P2X7 receptor resulted in the enhanced SIgA coating and enrichment of bacterial taxa that usually inhabit the small intestine *i*.*e*. *Lactobacillus*, *Enterococcus* and *Enterobacteriaceae*^23^; however, anti-inflammatory and anti-obesogenic taxa such as *Akkermansia* and *Prevotella*^24,25^ were depleted from the SIgA^+^ microbiota (LEfSe, *p* < 0.05, Wilcoxon rank-sum test, LDA > 2.0; Fig. 3a). *Lactobacillus* was the most abundant genus within the *P2rx7*^−/−^ IgA^+^ microbiota (mean relative abundance, 44.1% in *P2rx7*^−/−^, 14.1% in WT, Fig. 2b) and was highly enriched in this fraction (Fig. 3c). In the IgA^−^ microbiota of *P2rx7*^−/−^ mice, we observed a significant increase in the relative abundance of *Lachnospiraceae*, *Bilophila* and *Clostridia* (LEfSe, *p* < 0.05, Wilcoxon rank-sum test, LDA > 2.0; Fig. 3b), whereas the presorted faecal microbiota of *P2rx7*^−/−^ mice was depleted of bacterial taxa important for intestinal homeostasis, *e*.*g*. *Barnesiella*, *Ruminococcaceae*, *Clostridium* cluster *IV*^26,27^ (LEfSe, *p* < 0.05, Wilcoxon rank-sum test, LDA > 2.0; Fig. S2c). Notably, the genus *Turicibacter*, enriched in the IgA^−^ fraction of *P2rx7*^−/−^ mice (Fig. 3c), was exclusively present in these mice (Figs 2b and S2c). Altogether, these data suggest SIgA response in *P2rx7*^−/−^ mice conditions intestinal microbial ecology beyond bacterial taxa that are selectively targeted by SIgA.

### Correlation of gut microbes conditioned by enhanced SIgA response with metabolic and immunological parameters in *P2rx7*^−/−^ mice

**Figure 4:**
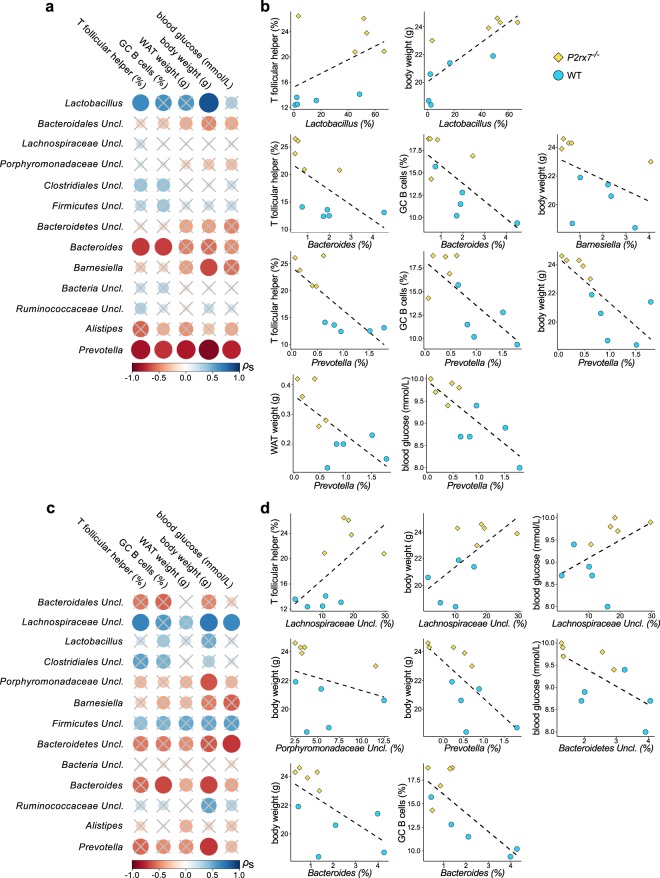
Alterations of metabolic and immunological parameters correlate with alterations of the gut microbiota in *P2rx7*^−/−^ mice. (**a**,**c**) Spearman’s *ρ* (*ρ*s) correlation between the relative abundance of the most represented bacterial genera (with relative abundance >0.5% and detectable in at least 70% of the samples) in the IgA^+^ (**a**) and IgA^−^ (**c**) fractions of faecal microbiota from WT and *P2rx7*^−/−^ mice with the indicated metabolic and immunological parameters. Solid circles represent the degree of correlation among the variables taken into account. Crossed circles indicate non-significant correlations; significant results with *p* < 0.05. (**b**,**d**) Correlation plots of the significant Spearman’s correlations between bacterial taxa and metabolic and immunological parameters of *P2rx7*^−/−^ and WT mice in the IgA^+^ (**b**) and IgA^−^ (**d**) fractions of the faecal microbiota. *P2rx7*^−/−^ and WT mice are represented as yellow diamonds and blue circles, respectively. Dashed lines indicate the regression curves.

The relationship between metabolic disorders and gut microbiota has been widely established^1^ as well as the role of the immune system and Tfh cells activity in selecting a beneficial microbiota for host metabolism^16^. The enhanced Tfh cells activity in *P2rx7*^−/−^ mice was accountable for gut microbiota alterations in both IgA^+^ and IgA^−^ fractions. To evaluate which bacterial taxa might be important for energy metabolism and regulation of mucosal immunity via P2X7, we correlated metabolic and immunologic parameters with the most abundant bacterial genera retrieved by IgA-SEQ. The genus *Lactobacillus*, within the IgA^+^ microbiota of *P2rx7*^−/−^ and WT mice, positively correlated with body weight and abundance of Tfh cells in PPs (Fig. 4a,b). On the other hand, we observed negative correlations of Tfh cells, GC B cells, blood glucose, body and WAT weight with the genera *Prevotella*, *Bacteroides* and *Barnesiella* (Fig. 4a,b). Thus, modified SIgA targeting of these genera by deregulated T follicular help in *P2rx7*^−/−^ mice could contribute to host metabolic alterations. Within the IgA^−^ microbiota of *P2rx7*^−/−^ and WT animals, the relative abundance of unclassified *Lachnospiraceae* positively correlated with body weight, blood glucose and Tfh cells while *Prevotella* and *Bacteroides* negatively correlated with body weight and % of GC B cells (Fig. 4c,d), consistent with previous observations on the high relative abundance of *Lachnospiraceae*, and low abundance of *Bacteroides* and *Prevotella* in obese individuals and mice^25,28,29^. Moreover, significantly increased *Lachnospiraceae* were found in the caecal microbiota of *P2rx7*^−/−^ mice^16^. Finally, different taxa (*i*.*e*. *Alistepes*, *Oscillibacter*, *Mucispirillum*, *Clostridium XIVb*, unclassified genera of *Clostridiales* and *Ruminococcaceae*) in the WT and *P2rx7*^−/−^ faecal microbiota negatively correlated with body and WAT weight, blood glucose and GC B cells (Fig. S2d,e). Altogether, these data suggest that P2X7 activity in Tfh cells conditions microbiota composition and host metabolism via regulated SIgA targeting of selected bacterial genera that in turn might affect metabolically relevant taxa independently of SIgA targeting.

### Intestinal *Lactobacillus* isolates from *P2rx7*^−/−^ mice alter glucose metabolism in wild-type animals

**Figure 5:**
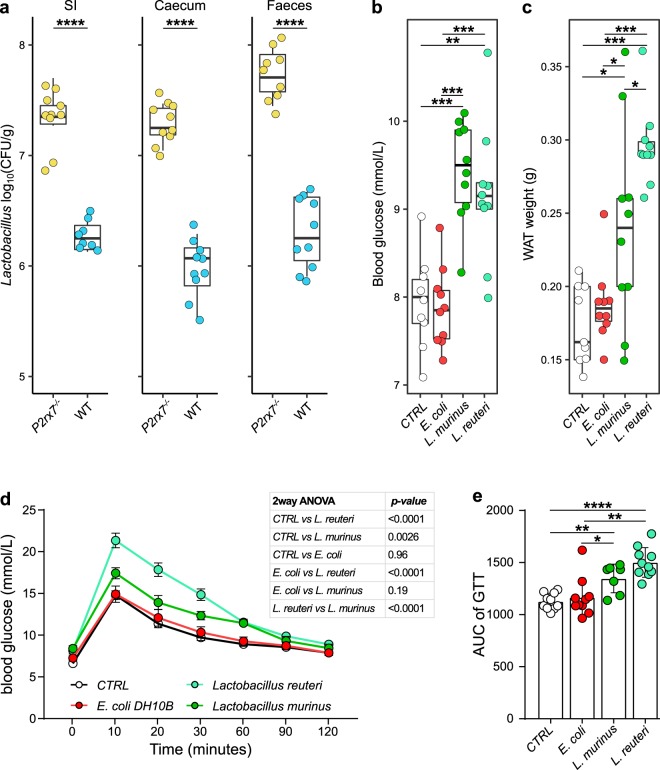
Altered glucose homeostasis and fat deposition in *Lactobacillus* treated WT animals. (**a**) Absolute quantification of the genus *Lactobacillus* by qPCR in the small intestine (SI), caecum and faeces of *P2rx7*^−/−^ (yellow) and WT (blue) mice. (**b**) Glycaemia after 21 days of treatment. (**c**) Fat deposition as measured by perigonadal white adipose tissue weight. (**d**) Glucose tolerance test (GTT). (**e**) Areas under the curve (AUC) of GTT. *p < 0.05, **p < 0.01, ***p < 0.001, ****p < 0.0001, Wilcoxon rank-sum test; n = 10 per group; n = 7 for GTT of *L*. *murinus* group.

The genus *Lactobacillus* was significantly enriched in the IgA^+^ fraction of the *P2rx7*^−/−^ microbiota (Fig. 3a,c) and correlated with the metabolic and immune phenotype of *P2rx7*^−/−^ mice (Fig. 4a,b). Different species of lactobacilli have been associated with body weight gain^30^ and juvenile growth rate^31^ through the increase of dietary protein digestion and amino acid intake by the host^32^. Quantification of the genus *Lactobacillus* by qPCR in samples from small intestine, caecum and faeces confirmed the significant enrichment of lactobacilli in the gut of *P2rx7*^−/−^ mice (Fig. 5a). To investigate whether lactobacilli may contribute to the metabolic alterations induced by non-functional P2X7 receptor^16^, we recovered from the gastrointestinal tract of *P2rx7*^−/−^ mice different isolates that were all belonging to the species *L*. *murinus* and *L*. *reuteri*. Accordingly, we detected increased titres of faecal IgA specific for these *Lactobacillus* species in *P2rx7*^−/−^ as compared to WT mice (Fig. S3c). Conversely, IgA coating of small intestine microbes by faecal IgA derived from either WT or *P2rx7*^−/−^ mice was undistinguishable (Fig. S3a,b), suggesting SIgA response in the small intestine of *P2rx7*^−/−^ mice is skewed toward lactobacilli.

We administered by oral gavage for three weeks the isolates *L*. *murinus* SI1/6 and *L*. *reuteri* SI1/3 from *P2rx7*^−/−^ mice to specific pathogen-free (SPF) mice that were depleted of endogenous microbiota by antibiotics (SPF-Abx). Treatment of SPF-Abx mice with both *Lactobacillus* isolates induced a significant increase of glycaemia compared to non-treated mice or animals gavaged with *E*. *coli* (Fig. 5b), altered glucose homeostasis with reduced glucose clearance in the glucose tolerance test (GTT) (Fig. 5d,e) as well as increased perigonadal fat deposition (Fig. 5c). The altered metabolic homeostasis observed in *Lactobacillus*-treated mice was unrelated to Tfh (% Tfh cells: CTRL, 7.77 ± 2.4; *E*. *coli* DH10B, 11.2 ± 4.2; *L*. *murinus*, 9.56 ± 2.1; *L*. *reuteri*, 9.43 ± 3.3) or GC B (% GC B cells: CTRL, 9.91 ± 2.4; *E*. *coli* DH10B, 9.82 ± 2.4; *L*. *murinus*, 11.8 ± 2.1; *L*. *reuteri*, 11.1 ± 1.9) cells abundance in PPs. Furthermore, oral gavage of *Lactobacillus* into *Igh-J*^−/−^ mice, which carry a deletion in the J region of the Ig heavy chain locus and lack SIgA, showed similar alterations in glucose metabolism to *Lactobacillus*-treated WT animals (Fig. S4) suggesting that SIgA were important in enriching *Lactobacillus* in the intestine of *P2rx7*^−/−^ mice but not necessarily required for inducing the observed metabolic alterations.

## Discussion

Intestinal homeostasis requires a balanced microbiota^1^, which is also shaped in structure and functions by secreted IgA^33^. IgA coating identifies bacterial taxa with the potential ability to interact with the host and colonize the intestinal mucosa; in addition, it can influence bacterial gene expression, metabolism and ability to colonize different intestinal ecological niches^8,9^. Since P2X7 deficiency leads to enhanced secretion of intestinal IgA and alterations of both gut microbiota and host metabolism^15,16^, the *P2rx7*^−/−^ mouse represents a unique model for the study of the role of SIgA in the remodelling of gut microbiota and metabolic homeostasis. In fact, the enhanced production of SIgA resulted in increased SIgA coating of bacteria typically residing in the small intestine, especially *Lactobacillus*, *Enterococcus* and *Enterobacteriaceae*^23^.

The *P2rx7*^−/−^ SIgA^+^ microbiota was characterized by a significant increase of the *Firmicutes/Bacteroidetes* ratio, a common feature of obese mice and humans^21,22^, suggesting that enhanced SIgA-coating could enrich bacterial taxa contributing to metabolic alterations. The genus *Lactobacillus*, belonging to the phylum *Firmicutes*, has been associated with body weight gain^30^, obesity^29^ and modulation of SIgA production^34^, although a consensus regarding its role in health and disease has not been fully achieved^35^. In *P2rx7*^−/−^ mice, we observed a positive correlation between the relative abundance of IgA^+^ *Lactobacillus* with body weight as well as with the abundance of Tfh cells in PPs. Consistent with a direct causal role of enriched lactobacilli in contributing to the metabolic phenotype of *P2rx7*^−/−^ mice, the administration of two *Lactobacillus* isolates from *P2rx7*^−/−^ to WT or *Igh-J*^−/−^ mice reproduced the impaired glucose metabolism observed in *P2rx7*^−/−^ mice. These experiments suggest that purinergic regulation of adaptive SIgA response in GALT can modulate intestinal colonization by commensals, which affect host physiology.

A physiological bacterial IgA coating regulated by T follicular regulatory (Tfr) cells and P2X7 proficient Tfh cells, contributes to the maintenance of a well-balanced intestinal microbial community within different ecological niches^11,16^. Specific changes in the IgA^+^ and IgA^−^ microbiota of *P2rx7*^−/−^ mice correlate with dysmetabolic features of these animals. How SIgA controls the diversification and balance of the gut microbiota is not yet clearly understood; our work sheds light on the importance of the regulation of T cell dependent SIgA via the eATP/P2X7 axis in controlling the abundance of bacterial taxa, such as *Lactobacillus*, that can affect host metabolic homeostasis.

In conclusion, by analysing mice deficient in the ATP-gated ionotropic P2X7 receptor, which limits Tfh cells in the PPs and adaptive SIgA production, we positively correlated Tfh cells number and body weight with increased SIgA coating and enrichment of lactobacilli. We hypothesize the eATP/P2X7 axis constitutes a crucial regulatory pathway in Tfh cells to ensure controlled SIgA coating and abundance of commensals which affect host metabolism.

## Materials and Methods

### Mice and *in vivo* experiments

C57BL/6J, *P2rx7*^−/−^ (B6.129P2-P2rx7tm1Gab/J) and *Igh-J*^−/−^ (B6.129P2-Igh-Jtm1Cgn/J) mice from Jackson Lab were bred in the specific pathogen-free (SPF) facility at the Institute for Research in Biomedicine, Bellinzona, Switzerland. The colonies of C57BL/6J, *P2rx7*^−/−^ and *Igh-J*^−/−^ were maintained onsite with heterozygous breeders and littermates kept in the same cages until weaning at 4 week of age. Animals were housed in ventilated cages in a 12 h light/dark cycle, with free access to water and standard autoclaved chow. Food intake was measured by using metabolic cages. For the *in vivo Lactobacillus* administration experiments, 4 weeks old C57BL/6J and *Igh-J*^−/−^ animals were treated with an antibiotic mixture containing Vancomycin (1.25 mg), Ampicillin (2.5 mg) and Metronidazole (1.25 mg) (VAM) in 200 µl water per mouse by oral gavage for 7 days to promote a more efficient bacterial colonization^36^. Later, these animals were given 5 * 10^9^ CFU of *Lactobacillus reuteri*, *Lactobacillus murinus* or *E*. *coli* DH10B by oral gavage in 200 µl PBS for 21 days. Glucose tolerance test was performed as follow: animals were fasted for 12 h and then received an intraperitoneal injection of glucose (2 g/kg of body weight). Blood glucose was measured using a glucometer (Healthpro-X1, Axapharm) on samples collected from tail vein. For RER measurement, mice were transferred to single housing in Phenomaster System (TSE Systems Gmbh, Bad Homburg, Germany) one day before the study start for acclimatization, followed by two days of continued measurements. During the study period, air flow, temperature, oxygen and carbon dioxide content, oxygen uptake (VO_2_), carbon dioxide production (VCO_2_) were measured simultaneously using standard indirect calorimetry analysis. Respiratory exchange ratio was calculated automatically from VO_2_ and VCO_2_. Data were collected in TSE Phenomaster software and exported to excel. For *ex vivo* experiments, mice were euthanized by CO_2_ inhalation and Peyer’s patches, white perigonadal adipose tissue and faeces, small intestine and caecal contents were collected. All animal experiments were performed in accordance with the Swiss Federal Veterinary Office guidelines and authorized by the relevant institutional committee (Commissione cantonale per gli esperimenti sugli animali) of the Cantonal Veterinary with authorization numbers TI44/18 and TI22/16.

### Cells isolation and flow cytometry

Single-cell suspensions were prepared from PPs harvested from the small intestine of C57BL/6J or *P2rx7*^−/−^ mice. Tfh and GC B cells were stained with labelled antibodies diluted in PBS with 2% heat-inactivated foetal bovine serum (FBS) for 20 min on ice. The following mouse antibodies (mAbs) were purchased from BD Biosciences (BD Biosciences, Franklin Lakes NJ, USA): biotin conjugated anti-CXCR5 (clone: 2G8, Cat.#: 551960), PE conjugated anti-Fas (clone: Jo2 Cat.#: 554258), PE conjugated anti-ICOS (clone: 7E.17G9, Cat.#: 552146). The following mAbs were purchased from Biolegend (Biolegend, San Diego, CA, USA): APC conjugated anti-PD-1 (Clone: RMPI-30, Cat.#: 109111), APC conjugated anti-B220 (clone: RA3-6B2, Cat.#: 103212), PE-Cy7 conjugated anti-CD4 (Clone: GK1.5, Cat.# 100422), APC-Cy7 conjugated anti-CD19 (clone: 6D5, Cat.#: 115530), APC conjugated streptavidin (Cat.#:405207). The following mAbs were purchased from eBioscience (eBioscience, Santa Clara, CA, USA): Percp-eFluor710 conjugated anti-CD3 (Clone: 17A2, Cat.#: 46-0032-80) and efluo405 conjugated streptavidin (Cat.#: 48-4317-82). Fluorescein labelled Peanut Agglutinin (PNA) (Cat.#: FL-10-71) was purchased from Vectorlabs (Vector Laboratories, Burlingame, CA, USA). Fluorescein Isothiocyanate (FITC) conjugated anti-IgA (Cat.#: 1040-02) and biotinylated anti-mouse IgA (Cat.#: 1040-08) were obtained from Southern Biotech. SYTO BC Green Fluorescent Nucleic Acid Stain (Cat.#: S34855) was purchased from Thermo Fisher Scientific. Samples were acquired on an LSRFortessa (BD Biosciences, Franklin Lakes NJ, USA) flow cytometer. Data were analysed using the FlowJo software (TreeStar, Ashland, OR, USA) or FACS Diva software (BD Biosciences, Franklin Lakes NJ, USA).

### Faecal IgA flow cytometry and sorting of IgA^+^ and IgA^−^ bacteria

For analysis of IgA coated bacteria in flow cytometry, fresh faecal pellets were collected into sterile 2 mL Eppendorf tubes and homogenized in PBS (0.1 g/ml). The homogenized samples were centrifuged at 400 × g for 5 min to remove larger particles from bacteria. Supernatants were centrifuged at 8,000 × g for 10 min to remove unbound IgAs. Bacterial pellets were resuspended in PBS 5% goat serum (Jackson Immunoresearch, West Grove, PA, USA), incubated 15 min on ice, centrifuged and resuspended in PBS 1% BSA for staining with APC conjugated rabbit anti-mouse IgA antibodies (Cat.#: SAB1186; Brookwood Biomedical, Birmingham, AL, USA). After 30 min incubation, bacteria were washed twice and resuspended in 2% paraformaldehyde in PBS for acquisition at LSRFortessa. Both for analysis and sorting of the IgA^+^ and IgA^−^ fractions at FACSAria, forward and side scatter parameters were used in logarithmic mode. SYTO BC was added to identify bacteria-sized particles containing nucleic acids. *Rag1*^−/−^ mice were used as control for absence of Igs-coated bacteria.

### Determination of binding of faecal IgA to small intestine microbiota and titers of faecal IgA specific for lactobacilli

Binding of faecal IgA to small intestine microbiota and titers of lactobacilli specific IgA in faecal samples were measured by flow cytometry. To detect the binding of faecal IgA to small intestine microbiota, the intestinal content of C57BL/6 and *P2rx7*^−/−^ mice was collected and homogenized in PBS (0.1 g/ml). The homogenized samples were centrifuged at 400 × g for 5 min to remove larger particles from bacteria. Supernatants were then centrifuged at 20,000 × g for 10 min to remove unbound IgAs. The pellet was resuspended in 1 ml PBS and 10 μl of bacterial suspension were incubated with 25 μl of fecal IgA from C57BL/6 or *P2rx7*^−/−^ mice at 4 °C for 1 h. After two washes, bacteria were incubated for 30 min with biotinylated anti-mouse IgA mAb followed by SYTO-BC and Alexa Fluor 405-labeled streptavidin. The samples were resuspended in 2% paraformaldehyde in PBS for acquisition on a FACSCanto using FSC and SSC parameters in logarithmic mode. To determine the titer of lactobacilli specific IgA in faecal samples, *L*. *reuterii* and *L*. *murinus* were resuspended at a density of 10^7^ bacteria ml^−1^. Fresh faecal samples were collected and carefully resuspended in PBS (0.01 g/ml). The obtained suspension was centrifuged two times at 20,000 × g and the supernatant collected to determine the titer of IgA specific for lactobacilli. Faecal samples were serially diluted and 25 μl of each dilution were incubated with 25 μl of bacterial targets suspension at 4 °C for 1 h. After two washes, bacteria were incubated for 30 min with monoclonal FITC anti-mouse IgA and then resuspended in 2% paraformaldehyde in PBS for acquisition on a FACSCanto using FSC and SSC parameters in logarithmic mode. ELISA was used to determine the total IgA concentration in an undiluted aliquot of the same faecal sample used for analysis in flow cytometry. Median fluorescence intensities (MFI) were plotted against IgA concentrations for each sample and 4-parameter logistic curves fitted using Prism (Graphpad, La Jolla, CA). Titers were calculated from these curves as the inverse of the antibody concentration giving an above-background signal. The concentration of total IgA titer required to achieve a given MFI (for example 200) was calculated by re-arrangement of the fitted 4-parameter logistic equation for each sample. As this value is low where a strong antibody response is present, the inverse of this value was plotted. Thus, titers are calculated as the inverse total antibody concentration required to achieve a given MFI. The y-axis value chosen as “above background” necessarily varies between experiments due to the flow cytometer settings, but is constant within any one analysis^37^.

### 16S rRNA gene sequencing and data analysis

DNA was extracted using the ZR faecal DNA Miniprep kit (Zymo Research, Irvine, CA, USA) following manufacturer’s instructions. A primer set specific for the V5–V6 hypervariable regions was used for the amplification of the bacterial 16S rRNA gene (Fw: 5′-ATTAGATACCCYGGTAGTCC-3′ and Rev: 5′-ACGAGCTGACGACARCCATG-3′)^38^. The 16S rRNA gene amplicons were then purified and pair-end sequenced on an Illumina MiSeq platform as previously described^39^. Illumina sequencing resulted in a total of 5,457,629 high quality reads with a mean of 181,921 ± 35,928 sequences *per* sample. The raw fastq files were submitted to the European Nucleotide Archive with accession number PRJEB20647 (http://www.ebi.ac.uk/ena/data/view/PRJEB20647). Sample accession IDs and metadata, unrarefied OTU table and taxonomic classifications are available in the Table S1. Reads were pre-processed using the MICCA pipeline (v1.5.0) (http://www.micca.org)^40^. The overlapping 2 × 250 paired-end reads were merged using micca mergepairs^41^. Forward and reverse primer trimming and quality filtering were performed using micca trim and micca filter, respectively. *De novo* greedy clustering and chimera filtering were performed by using micca otu: operational taxonomic units (OTUs) were assigned by clustering the sequences with a threshold of 97% pairwise identity, and their representative sequences were taxonomically classified using micca classify with the RDP classifier version 2.11^42^. Singleton OTUs and OTUs present only in the sorted IgA^+^ and IgA^−^ fractions but not in the pre-sorted faecal samples were discarded from the final OTU table. Multiple sequence alignment was performed using the Nearest Alignment Space Termination (NAST)^43^ algorithm implemented in micca msa with the template alignment clustered at 97% similarity of the Greengenes database^44^ (release 13_05). The phylogenetic tree was inferred using micca tree^45^. Sampling heterogeneity was reduced rarefying samples at the depth of the less abundant sample (56,444 sequences). *Alpha*- (within-sample richness) and *beta*-diversity (between-sample dissimilarity) estimates were computed using the *phyloseq* R package^46^. Permutational MANOVA (PERMANOVA) was performed on the unweighted UniFrac distance and Bray-Curtis dissimilarity using the adonis() function of the *vegan* R package with 999 permutations. The identification of taxa differentially distributed in the groups of study was obtained by using the linear discriminant effect size analysis (LEfSe)^47^. LEfSe ranks features by effect size, putting at the top features that explain most of the biological difference. LEfSe combines Kruskal-Wallis and Wilcoxon rank-sum tests with linear discriminant analysis (LDA). LEfSe was performed under the following conditions: α value for the statistical test equal to 0.05 and threshold on the logarithmic LDA score for discriminative features equal to 2.0. Spearman’s correlation tests were computed using the *psych* R package^48^. All statistical analyses were performed using R^49^ and GraphPad Prism v7.04 (GraphPad Software, La Jolla, CA, USA). A *p*-value < 0.05 was considered significant in all cases.

### Quantitative PCR of intestinal lactobacilli

Quantification of *Lactobacillus* in faeces, small intestine and caecal contents was achieved by using the Fast SYBR™ Green Master Mix (Applied Biosystems™, Waltham, MA, USA) with the QuantStudio 3 Real-Time PCR System (Applied Biosystems™, Waltham, MA, USA). The PCR reaction mix contained 1X Fast SYBR™ Green Master Mix, 0.4 μM of each *Lactobacillus* specific primer (F_alllact_IS: TGG ATG CCT TGG CAC TAG GA; R_alllact_IS: AAA TCT CCG GAT CAA AGC TTA CTT AT)^50^ and 20 ng of gDNA as template. A seven point standard curve consisting in tenfold serial dilutions of gDNA extracted from a *Lactobacillus* pure culture at known concentration was used for absolute quantification. Amplification specificity was checked by melting curve analysis, efficiency and reliability of PCR amplifications were also calculated.

### Isolation of intestinal *Lactobacillus spp*. and bacterial cultures

Fresh faeces, small intestinal and caecal contents were collected from *P2rx7*^−/−^ mice and resuspended 1:10 (weight: volume) in PBS +0.1% L-cysteine-HCl. The suspensions have been then mixed and tenfold serially diluted. The dilutions were plated on LAMVAB medium^51^ and incubated at 37 °C under anaerobic conditions (AnaeroGen, Oxoid) in jars (AnaeroJar, Oxoid) for 72 h. Based on the identification of different colony morphotypes, 72 isolates have been picked, re-isolated on LAMVAB medium in order to obtain pure colonies and identified by Sanger sequencing of the 16S rRNA gene (8F: AGA GTT TGA TCC TGG CTC AG; 1391R: GAC GGG CGG TGT GTR CA). The *Lactobacillus* isolates were grown in *Lactobacillus*-MRS broth (EMD Millipore, Burlington, MA, USA) at 37 °C under anaerobic conditions. *E*. *coli* DH10B was grown aerobically in Luria-Bertani broth (Sigma-Aldrich, Saint Louis, MO, USA) at 37 °C.

### Accession codes

Raw sequences are available in the European Nucleotide Archive (ENA) with Accession Number PRJEB20647 (http://www.ebi.ac.uk/ena/data/view/PRJEB20647). Sample metadata, unrarefied OTU table and taxonomic classifications are available in the Table S1.

## Supplementary information

Supplementary information

Supplementary Table 1
