# Supplementary material for: Enrichment of intestinal Lactobacillus by enhanced secretory IgA coating alters glucose homeostasis in P2rx7−/− mice
Source: Sci Rep. 2019 Jun 27;9:9315. doi: 10.1038/s41598-019-45724-9 (PMC6597561; doi:10.1038/s41598-019-45724-9)
Supplement: Supplementary file 1 — Supplementary information [file 41598_2019_45724_MOESM1_ESM.pdf]

Enrichment of intestinal *Lactobacillus* by enhanced secretory IgA coating alters glucose homeostasis in *P2rx7*$^{-/-}$ mice

Lisa Perruzza, Francesco Strati, Giorgio Gargari, Anna Maria D'Erchia, Bruno Fosso, Graziano Pesole, Simone Guglielmetti, Fabio Grassi

Supplementary information

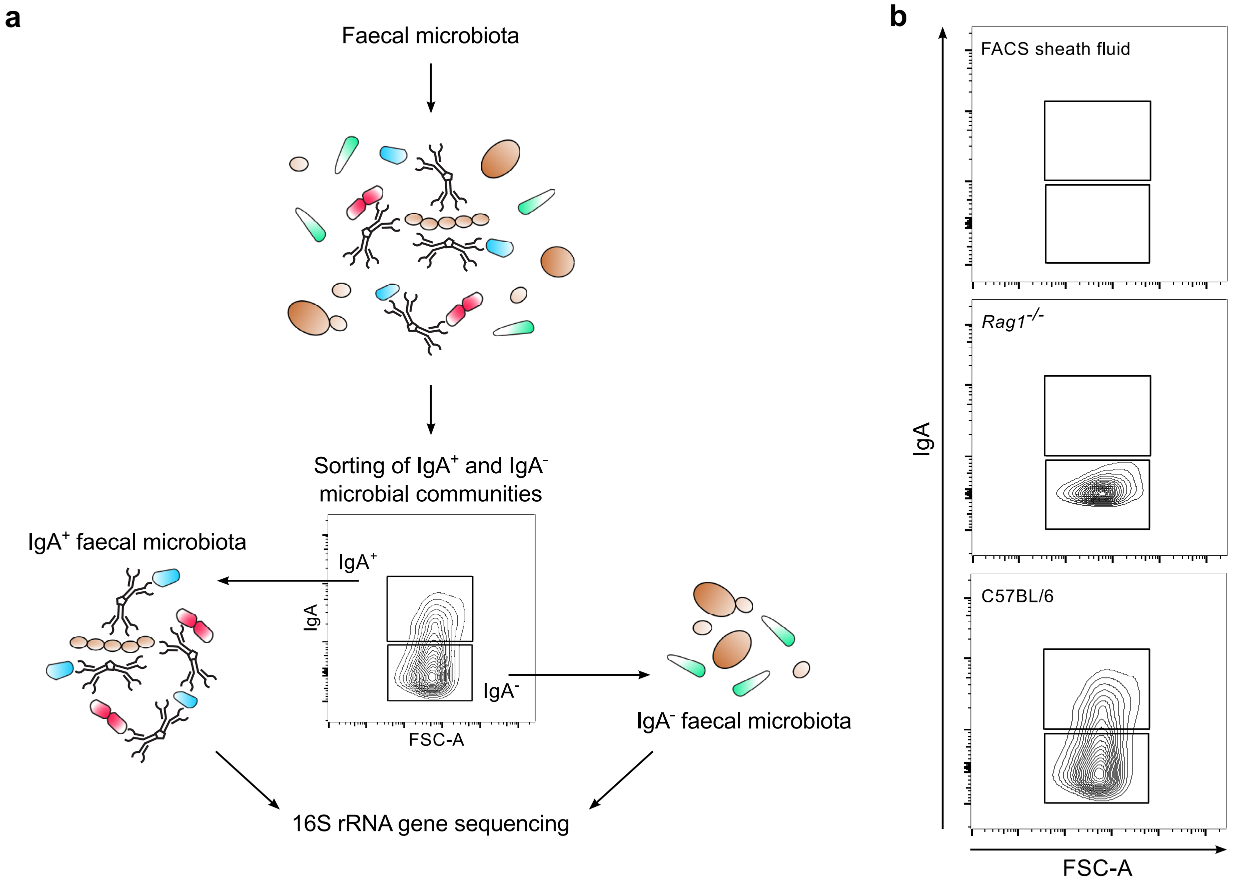

Supplementary Figure S1. IgA-SEQ analysis. a) Workflow for the characterization of the IgA$^+$ and IgA$^-$ fractions of the faecal microbiota. From faecal samples, IgA-coated (IgA$^+$) bacteria were separated from non-coated (IgA$^-$) bacteria by flow cytometry and subsequently characterized by 16S rRNA gene sequencing. **b)** Representative contour plots for IgA coating and forward scatter (FSC-A) of faecal bacteria isolated from the indicated mice and FACS sheath fluid used as negative control.

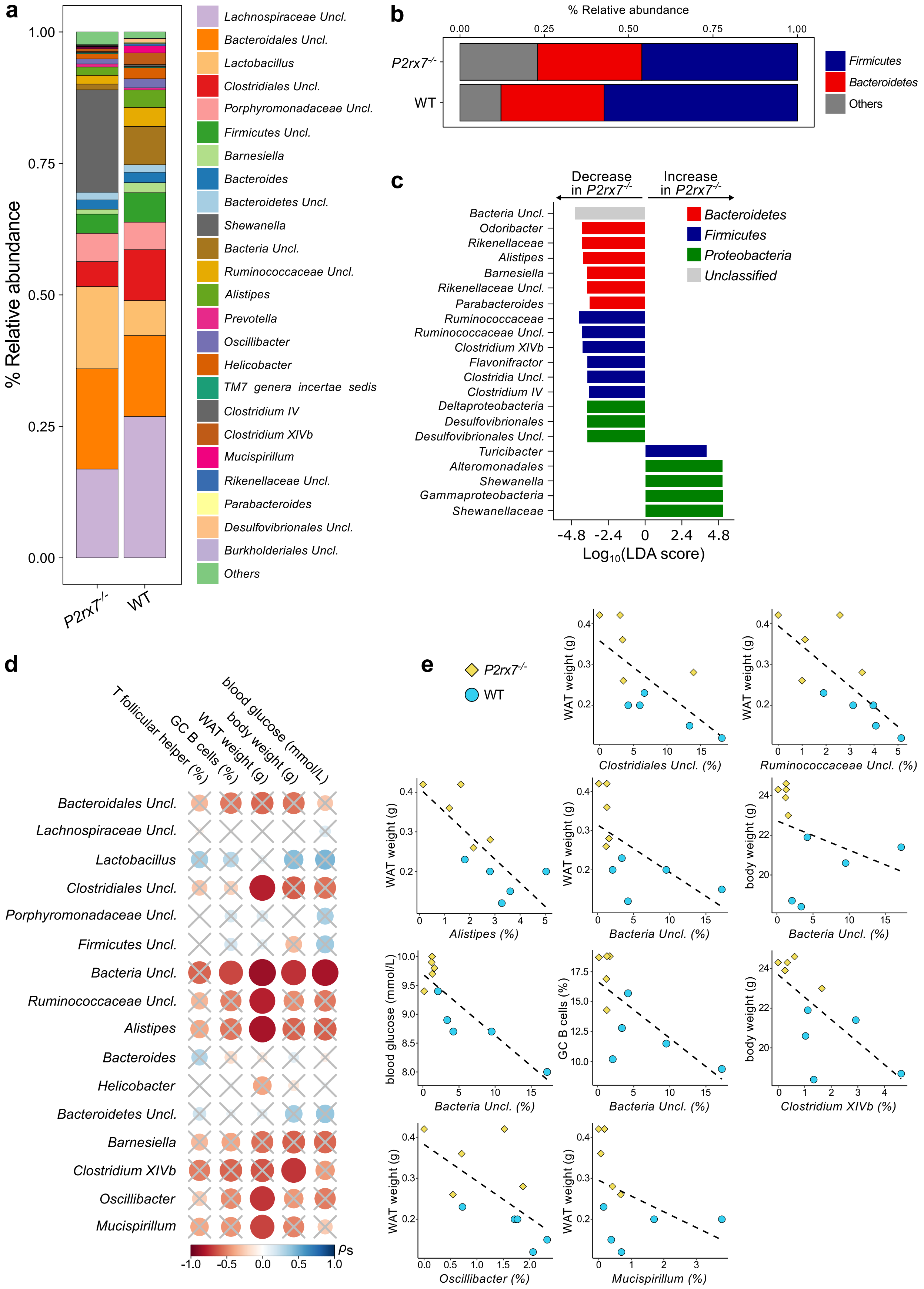

a
% Relative abundance
1.00
0.75
0.50
0.25
0.00
P2rx7-/-
WT
Lachnospiraceae Uncl.
Bacteroidales Uncl.
Lactobacillus
Clostridiales Uncl.
Porphyromonadaceae Uncl.
Firmicutes Uncl.
Barnesiella
Bacteroides
Bacteroidetes Uncl.
Shewanella
Bacteria Uncl.
Ruminococcaceae Uncl.
Alistipes
Prevotella
Oscillibacter
Helicobacter
TM7 genera incertae sedis
Clostridium IV
Clostridium XIVb
Mucispirillum
Rikenellaceae Uncl.
Parabacteroides
Desulfovibrionales Uncl.
Burkholderiales Uncl.
Others
b
% Relative abundance
0.00
0.25
0.50
0.75
1.00
P2rx7-/-
WT
Firmicutes
Bacteroidetes
Others
c
Decrease in P2rx7-/-
Increase in P2rx7-/-
Bacteria Uncl.
Odoribacter
Rikenellaceae
Alistipes
Barnesiella
Rikenellaceae Uncl.
Parabacteroides
Ruminococcaceae
Ruminococcaceae Uncl.
Clostridium XIVb
Flavonifractor
Clostridia Uncl.
Clostridium IV
Deltaproteobacteria
Desulfovibrionales
Desulfovibrionales Uncl.
Turicibacter
Alteromonadales
Shewanella
Gammaproteobacteria
Shewanellaceae
Bacteroidetes
Firmicutes
Proteobacteria
Unclassified
-4.8
-2.4
0
2.4
4.8
Log10(LDA score)
d
T follicular helper (%)
GC B cells (%)
WAT weight (g)
body weight (g)
blood glucose (mmol/L)
Bacteroidales Uncl.
Lachnospiraceae Uncl.
Lactobacillus
Clostridiales Uncl.
Porphyromonadaceae Uncl.
Firmicutes Uncl.
Bacteria Uncl.
Ruminococcaceae Uncl.
Alistipes
Bacteroides
Helicobacter
Bacteroidetes Uncl.
Barnesiella
Clostridium XIVb
Oscillibacter
Mucispirillum
ρs
-1.0
-0.5
0.0
0.5
1.0
e
P2rx7-/-
WT
WAT weight (g)
Clostridiales Uncl. (%)
Ruminococcaceae Uncl. (%)
Alistipes (%)
Bacteria Uncl. (%)
body weight (g)
blood glucose (mmol/L)
GC B cells (%)
Clostridium XIVb (%)
Oscillibacter (%)
Mucispirillum (%)

Supplementary Figure S2: Characterization of the faecal microbiota from WT and *P2rx7*$^{-/-}$ mice. **a)** Mean relative abundance, at genus level, of the presorted faecal microbiota of *P2rx7*$^{-/-}$ and WT mice. All bacterial genera with relative abundance <0.1% are reported together and labelled as "others". **b)** Mean relative abundance (%) of *Firmicutes* and *Bacteroidetes* in the presorted faecal microbiota of *P2rx7*$^{-/-}$ and WT mice. The total abundance of all other phyla is reported as "others". **c)** Log_{10} of LDA scores for the most discriminant bacterial taxa identified by LEfSe in the presorted faecal microbiota of *P2rx7*$^{-/-}$ and WT mice. Positive and negative LDA scores indicate the taxa enriched or depleted in *P2rx7*$^{-/-}$ mice. Only taxa having a $p<0.05$ (Wilcoxon rank-sum test) and LDA>|2.0| are shown. **d)** Spearman's ρ (ρs) correlation between the relative abundance of the most abundant bacterial genera (>0.5% and detectable in at least 70% of the samples) in the presorted faecal microbiota of *P2rx7*$^{-/-}$ and WT mice with metabolic and immunological parameters. **e)** Correlation plots of the significant Spearman's correlations between bacterial taxa and metabolic and immunological parameters of *P2rx7*$^{-/-}$ and WT mice. *P2rx7*$^{-/-}$ and WT mice are coloured in yellow and blue and shaped as diamonds and circles, respectively. Dashed lines indicate the regression curves.

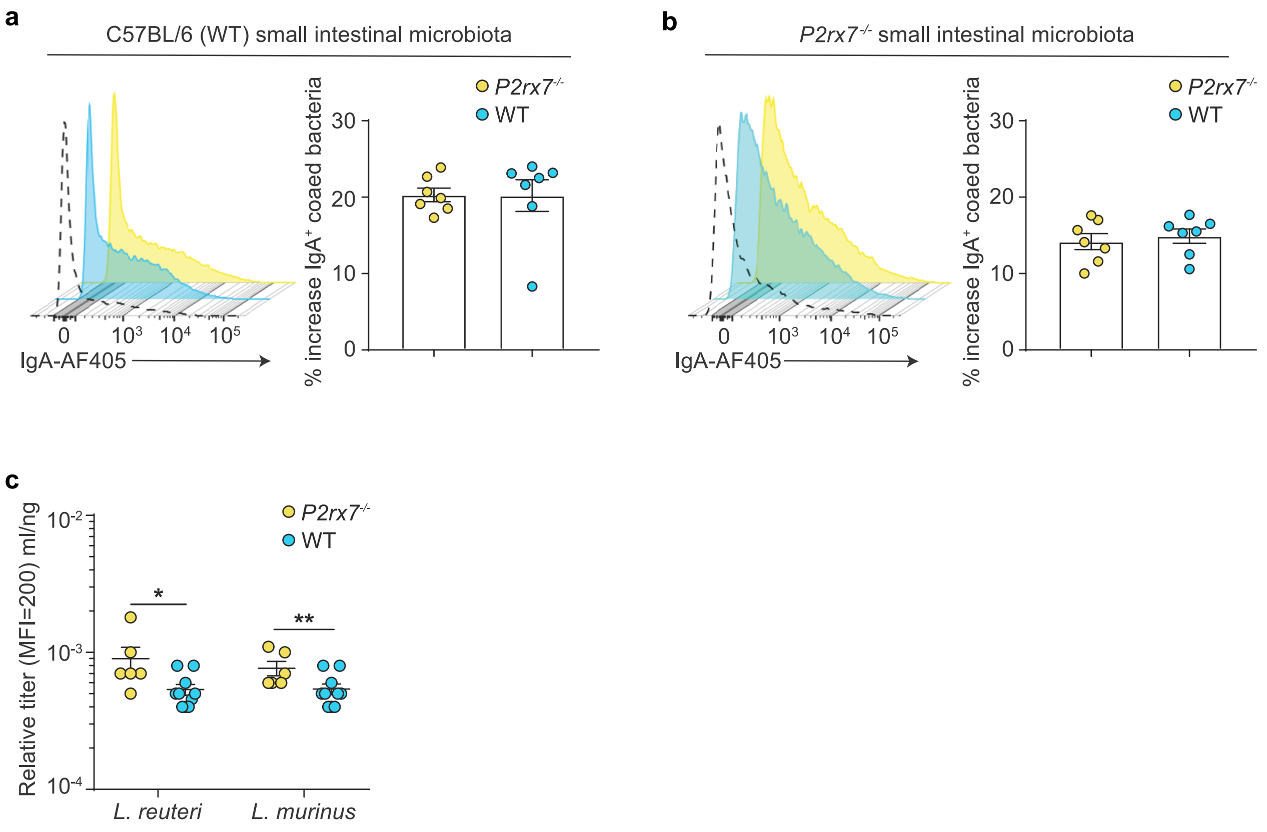

Supplementary Figure S3: Selective targeting of *Lactobacillus* by SIgA in *P2rx7*$^{-/-}$ mice. Flow cytometry profiles with AF405-labelled anti-mouse IgA and statistics of relative increase in IgA coating of small intestine microbiota from **a)** WT or **b)** *P2rx7*$^{-/-}$ mice by faecal IgA from WT (light blue graph) or *P2rx7*$^{-/-}$ (yellow graph) mice**. c)** Faecal anti-*L. reuteri* and anti-*L. murinus* IgA titers in WT and *P2rx7*$^{-/-}$ mice.

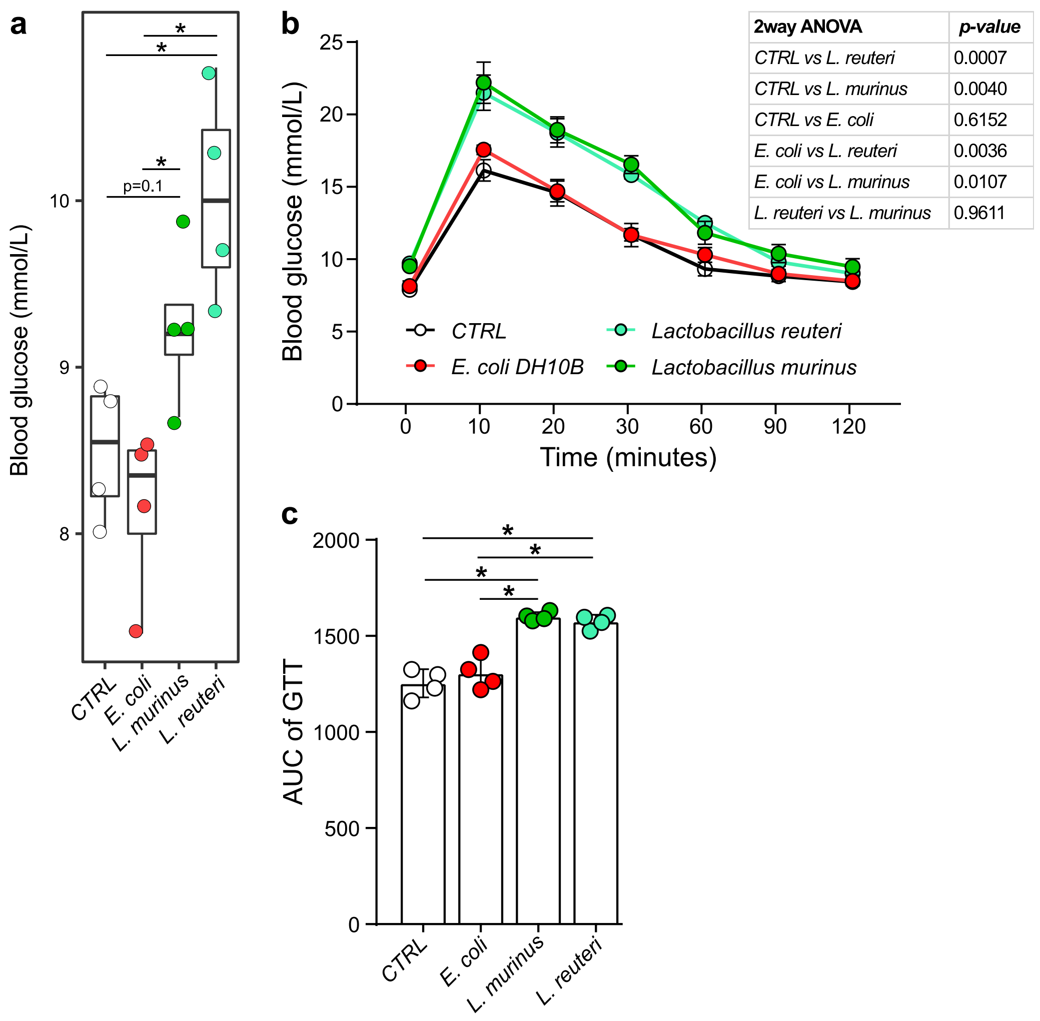

Supplementary Figure S4: Altered glucose homeostasis in *Lactobacillus* treated *Igh-J*$^{-/-}$ animals. a) Glycaemia after 21 days of treatment. **b)** Glucose tolerance test. **c)** Areas under the curve (AUC) of GTT. **p*<0.05, Wilcoxon rank-sum test; n=4 per group.

Supplementary Table S1: Sample metadata, unrarefied OTU table and taxonomic classification.
